# Risk of Breast Cancer by Type of Menopausal Hormone Therapy: a Case-Control Study among Post-Menopausal Women in France

**DOI:** 10.1371/journal.pone.0078016

**Published:** 2013-11-01

**Authors:** Emilie Cordina-Duverger, Thérèse Truong, Antoinette Anger, Marie Sanchez, Patrick Arveux, Pierre Kerbrat, Pascal Guénel

**Affiliations:** 1 Inserm (Institut National de la Santé et de la Recherche Médicale), CESP (Centre de recherche en Epidémiologie et Santé des Populations), Unit 1018, Villejuif, France; 2 University Paris-Sud, UMRS 1018, Villejuif, France; 3 Centre Georges-François Leclerc, Breast Cancer Registry of Côte d’Or, Dijon, France; 4 Centre Eugène Marquis, Rennes, France; University of North Carolina School of Medicine, United States of America

## Abstract

**Background:**

There is extensive epidemiological evidence that menopausal hormone therapy (MHT) increases breast cancer risk, particularly combinations of estrogen and progestagen (EP). We investigated the effects of the specific formulations and types of therapies used by French women. Progestagen constituents, regimen (continuous or sequential treatment by the progestagen), and time interval between onset of menopause and start of MHT were examined.

**Methods:**

We conducted a population-based case-control study in France in 1555 menopausal women (739 cases and 816 controls). Detailed information on MHT use was obtained during in-person interviews. Odds ratios and 95% confidence interval adjusted for breast cancer risk factors were calculated.

**Results:**

We found that breast cancer risk differed by type of progestagen among current users of EP therapies. No increased risk was apparent among EP therapy users treated with natural micronized progesterone. Among users of EP therapy containing a synthetic progestin, the odds ratio was 1.57 (0.99-2.49) for progesterone-derived and 3.35 (1.07-10.4) for testosterone-derived progestagen. Women with continuous regimen were at greater risk than women treated sequentially, but regimen and type of progestagen could not be investigated independently, as almost all EP combinations containing a testosterone-derivative were administered continuously and vice-versa. Tibolone was also associated with an increased risk of breast cancer. Early users of MHT after onset of menopause were at greater risk than users who delayed treatment.

**Conclusion:**

This study confirms differential effects on breast cancer risk of progestagens and regimens specifically used in France. Formulation of EP therapies containing natural progesterone, frequently prescribed in France, was not associated with increased risk of breast cancer but may poorly protect against endometrial cancer.

## Introduction

Menopausal hormone therapy (MHT) has been prescribed for more than fifty years to prevent discomfort caused by the menopause. When MHT started to be used, most preparations contained estrogen alone. In the 1970s, it was shown that the use of estrogen-alone therapy was associated with risk of endometrial cancer [1,2]. Progestagens were then added to the preparations to oppose the effects of estrogens and prevent endometrial cancer [3,4]. In 2002, the Women’s Health Initiative (WHI), a randomized clinical trial, reported that the use of combined estrogen-progestagen therapy was associated with an increased incidence of breast cancer [5]. This publication led to a dramatic decrease of EP treatment sales [6-9]. Today, there is considerable epidemiologic evidence that menopausal exposure to exogenous sex steroid hormones plays an important role in the development of breast cancer in women and combined EP hormonal therapy has been classified as carcinogenic to humans (Group 1) [10].

There is a large variety of MHTs available around the world, with country-specific types of molecules and regimens (continuous supply of progestagen or sequential). It has been shown that users of combined estrogen-progestin MHT have a higher risk of breast cancer than estrogen-only users, that the risk is particularly elevated in current users and that it increases with duration of use [11,12]. Fixed combinations with a continuous supply of progestagen were associated with a greater risk of breast cancer than sequential combinations [13]. Since different progestagens act differently on hormone receptors and have diverse biological effects [14], the question arises on whether different progestagens are associated with risk variations. It has also been reported that a short time interval between onset of menopause and start of MHT may influence breast cancer risk [15-17]. Because MHT prescriptions are strongly dependent on the particular country in which they are made, direct extrapolations of MHT-associated risk between studies conducted in different populations are difficult.

In combined EP therapies used in France, the estrogen component is usually estradiol, but preparations may contain a large variety of progestagens [18,19]. While micronized progesterone has been commonly prescribed in France, it has rarely been used in other countries [13,18]. Chemically derived progestagens in EP therapies are most often progesterone-derivatives [18], while testosterone-derivatives, usually prescribed in Northern European countries such as Denmark, Norway, UK or Germany, has been used more rarely in France [13,18,20]. In addition, tibolone a synthetic hormone licensed in the 1990s, with estrogenic and progestogenic properties, has been prescribed since 2000.

Data on breast cancer risk associated with MHT use in France are based primarily on the E3N prospective cohort study of French female teachers [21]. It was reported in this study that breast cancer risk was increased in current users of EP therapy containing synthetic progestagens, but not in users of EP therapy containing natural micronized progesterone. This finding could not be replicated in independent studies. It has also been hypothesized that breast cancer risk may differ by type of synthetic progestagen (derived from progesterone or from testosterone) but no clear difference between testosterone- and progesterone-derivatives have been reported [13,21,22]. As for tibolone, it was recently suspected to play a role in breast cancer [13,22,23], but results have not been consistent across studies [20,24,25]. 

In the present study, we aimed at examining breast cancer risk by type of MHT used in France from the data of a large population-based case-control study. We were interested in particular in the type of progestagen, regimen and delay between onset of menopause and start of therapy. 

## Methods

Ethics statement: The study was approved by the Ethic Committee of Kremlin-Bicêtre, France (Jan 2005) and by the National Data Protection Agency (Dec 2004). All participants signed informed consent.

The CECILE study is a population-based case-control study in *Côte d'Or* and in *Ille-et-Vilaine*, two French administrative areas (*départements*) located in Eastern and Western part of France, respectively. 

### Recruitment of cases and controls

The case group included incident cases of in situ or invasive breast cancer diagnosed between April 2005 and March 2007 in women aged 25-75 who resided in one of the two study areas. Patients were recruited in the main cancer hospital in each area (Centre Eugène Marquis in Rennes and Centre Georges-François Leclerc in Dijon), as well as from smaller public and private hospitals that also recruited breast cancer patients. Among the 1553 eligible cases identified during the study period, 163 refused to participate, 151 could not be contacted, and 7 died before the interview, leaving 1232 cases included in the study (participation 79.3%).

Controls were women without a previous history of breast cancer recruited in the general population, and frequency-matched to the cases by 10-year age group and study area. To select the controls, we contacted a random sample of private homes by telephone. Phone numbers were selected from the telephone directory where unlisted numbers had previously been re-created. If a woman was living in the residence reached by telephone, she was invited to participate in the study within predefined quotas by age and socio-economic status (SES). Quotas by age were applied to obtain similar distributions by age among controls and among cases (frequency-matching). Quotas by SES in control women were applied to reflect the distribution by SES of women in the general population in each study area, conditionally to age. Using predefined proportion of controls by SES was used to prevent selection biases that could arise from differential participation rates across SES categories. Among 1731 controls identified by telephone fulfilling eligibility criteria, 260 declined participation for an in-person interview and 154 could not be re-contacted, leaving 1317 women available for the study (participation 76.1%). 

### Selection of study subjects in the present analysis

Only menopausal women were included in the analysis. Women were considered menopausal if they had had no menstruation for twelve months or more (natural menopause, n=936), if they had bilateral oophorectomy (artificial menopause, n=93), or if they used MHT before natural cessation of menstruation (n=352). Women with unknown menopausal status (n=199), because of hysterectomy before cessation of menstruations or unknown date of last menstruation, were considered menopausal if they were 50 years old or more (the median age at menopause in women with natural menopause, n=174). Women with unknown menopausal status below 50 years old were excluded from the analysis (n=25). In total, the study included 1555 women, 739 cases and 816 controls (table 1).

**Table 1:** Distribution of cases and controls in menopausal women according to selected characteristics and risk factors for breast cancer.

	**Cases**		**Controls**			
	**(n=739)**		**(n=816)**		**OR^a^**	**95% CI**
	**N**	**%**	**N**	**%**		
**Study area (Département)**						
Côte d'Or	235	31.8	285	34.9		
Ille et Vilaine	504	68.2	531	65.1		
**Age (years)**						
35-44	1	0.1	2	0.2		
45-54	121	16.4	142	17.4		
55-64	347	47.0	356	43.6		
65-74	270	36.5	316	38.7		
**Personal history of benign breast disease**						
no	428	57.9	549	67.4	1	ref
yes	311	42.1	266	32.6	**1.45**	**[1.16-1.80]**
**Family history of breast cancer in first degree relatives**						
no	604	81.7	716	87.7	1	ref
yes	135	18.3	100	12.3	**1.56**	**[1.17-2.09]**
**Height at 20 years old (cm)**						
≤ 155	126	17.2	138	17.2	1	ref
]155-160]	221	30.2	249	31.1	0.98	[0.71-1.34]
]160-170]	332	45.4	372	46.4	1.06	[0.78-1.43]
>170	53	7.2	42	5.2	1.47	[0.89-2.41]
**Body Mass Index (kg/m^2^)**						
<18,5	16	2.2	21	2.6	0.74	[0.37-1.48]
18.5-25	371	50.5	411	50.4	1	ref
25-30	226	30.7	241	29.6	1.06	[0.84-1.36]
>30	122	16.6	142	17.4	1.04	[0.77-1.40]
**Age at menarche (years)**						
≤ 11	131	18.0	122	15.1	1	ref
12	179	24.7	172	21.3	1.03	[0.73-1.44]
13	155	21.3	174	21.6	0.86	[0.91-1.21]
14	143	19.7	165	20.5	0.84	[0.58-1.20]
≥ 15	118	16.3	173	21.5	**0.69**	**[0.48-0.99]**
**Parity**						
Nulliparous	79	10.7	50	6.1	1	ref
1 FTP	109	14.7	113	13.8	**0.49**	**[0.29-0.81]**
2 FTP	279	37.8	270	33.1	**0.59**	**[0.37-0.92]**
3 FTP	183	24.8	245	30.0	**0.46**	**[0.29-0.73]**
≥ 4 FTP	89	12.0	138	16.9	**0.43**	**[0.26-0.71]**
**Age at first full-term pregnancy (among parous women**)						
<22 yrs	185	28.0	253	33.0	1	ref
22-24 yrs	196	29.7	252	32.9	1.01	[0.77-1.34]
25-27 yrs	141	21.4	167	21.8	1.06	[0.78-1.45]
>27 yrs	138	20.9	94	12.3	**1.84**	**[1.29-2.63]**
**Breast-feeding (among parous women)**						
never	349	53.5	400	52.3	1	ref
<26 weeks	224	34.4	261	34.1	1.02	[0.80-1.31]
26-52 weeks	54	8.3	67	8.8	1.06	[0.71-1.59]
>52 weeks	25	3.8	37	4.8	0.97	[0.55-1.71]
**Oral contraceptive use**						
never	344	46.5	336	41.2	1	ref
ever	395	53.5	479	58.8	0.81	[0.64-1.03]
**Age at menopause (natural menopause or due to ovariectomy)**						
<48	105	22.7	115	22.9	1	ref
48-50	136	29.4	157	31.2	0.92	[0.63-1.35]
51-53	133	28.8	126	25.0	1.07	[0.72-1.58]
≥ 54	88	19.0	105	20.9	0.96	[0.65-1.43]

a Odds Ratios adjusted for age, study area and all other variables in the table

### Data Collection

Data pertaining to each study subject were obtained from a structured questionnaire during in-person interviews conducted by trained interviewers. We elicited information on sociodemographic characteristics, history of previous diseases, family history of cancer, menstruations, oral contraceptives, infertility, reproductive history, residential and occupational history, lifetime consumption of alcohol and tobacco, recreational activities, and dietary habits. A blood sample was collected for each case and control to collect DNA and serum samples. For each MHT, we obtained information on name, dates of start and end of use. To help women remember drug names during the interview, they were given a list of fifty MHTs commonly prescribed in France. 

Information on in situ or invasive tumor type, histology (ductal, lobular, other), estrogen (ER) and progesterone receptor (PR) status was obtained from the pathology report. 

We classified women according to the time since last use of MHT. Current users were women treated by MHT at reference date (date of diagnosis for the cases and date of interview for the controls) or who stopped treatment less than one year before reference date.

MHTs were classified in estrogen-only therapy, EP therapy, and tibolone. EP therapy was subdivided in 3 subtypes according to the progestagen constituent: natural micronized progesterone, progesterone-derivatives, and testosterone-derivatives. 

We also determined the time interval between age at onset of menopause and age at first use of MHT. For women who used MHT before cessation of menstruations, the time interval was set to zero. Menopausal women with unknown age at menopause and/or unknown age at first use of MHT (17 cases and 10 controls) were excluded from these analyses. 

### Statistical analysis

The odds ratios (OR) and their 95% confidence intervals were estimated using unconditional logistic regression models. Polytomous logistic regression was also used in the analyses where the case group was subdivided according to histological type (ductal, lobular) and according to the estrogen and progesterone receptor status.

All analyses were adjusted for age (5-year groups) and study area. We also adjusted for the following breast cancer risk factors: age at menarche (≤ 11, 12, 13, 14, ≥ 15 years), parity (0, 1, 2, 3, ≥ 4), age at first full-term pregnancy (<22, 22-24, 25-27, ≥ 28 years), duration of breast-feeding (0, <26, 26-52, >52 weeks), oral contraceptive use (ever/never), personal history of benign breast disease (yes/no), family history of breast cancer (yes/no) and body mass index (BMI) (<18.5, [18.5-25[ [25-30[,, ≥30 kg/m^2^).

All analyses were conducted using SAS computer software (version 9.2, Cary, North Carolina).

## Results

The distribution of the 739 cases and 816 controls by socio-demographic characteristics and selected breast cancer risk factors are shown in table 1. Due to frequency-matching, distributions by study area and age were similar in the two groups. Breast cancer risk increased in women with a history of benign breast disease and a family history of breast cancer in first-degree relatives. Increased risk was also seen in women with early age at menarche, low parity, late age at first full-term pregnancy. Cases and controls did not differ with respect to height, body mass index, alcohol or tobacco consumption, duration of breast-feeding and age at menopause.

Odds ratios for breast cancer by type and duration of MHT use are shown in table 2 for past and current users separately. Among current users, the odds ratio was 1.19 (0.69-2.04) for estrogen-only and 1.33 (0.92-1.92) for EP therapy users. The odds ratio increased to 1.55 (1.02-2.36) in current users of EP combinations treated for 4 or more years. Current use of tibolone was also associated with elevated odds ratios that did not reach statistical significance, and that increased with duration of use (p trend=0.07). Women who used MHT in the past were not at increased risk of breast cancer as compared to never users. Only current MHT users will be considered in subsequent analyses.

**Table 2:** Odds ratios for breast cancer by type of menopausal hormone therapy and duration of use in current and past users.

	**Current users**				**Past users**			
**Duration of MHT use**	**Cases**	**Controls**	**OR^a^**	**95% CI**	**Cases**	**Controls**	**OR^a^**	**95% CI**
**Never**	311	357	1	ref	311	357	1	ref
**Estrogen-only therapy**								
Any duration	34	31	1.19	[0.69-2.04]	72	93	0.83	[0.57-1.21]
< 4 years	14	10	1.58	[0.67-3.75]	26	32	0.90	[0.51-1.59]
≥ 4 years	20	20	1.01	[0.51-2.02]	39	53	0.77	[0.48-1.24]
**Combined EP therapy**								
Any duration	92	82	1.33	[0.92-1.92]	133	171	0.78	[0.57-1.05]
< 4 years	17	26	0.86	[0.43-1.73]	25	38	0.65	[0.37-1.14]
≥ 4 years	73	56	**1.55**	**[1.02-2.36]**	101	129	0.80	[0.57-1.12]
**Tibolone**								
Any duration	17	8	2.42	[0.96-6.10]	10	15	0.55	[0.22-1.36]
< 4 years	7	5	2.04	[0.59-7.07]	5	11	0.46	[0.15-1.42]
≥ 4 years	10	3	3.09	[0.79-12.0]	4	4	0.66	[0.13-3.26]

a Odds Ratios adjusted for Study area / Age at reference date/ Age at menarche / Parity / Age at first full-term pregnancy / Breast feeding /History of benign breast disease / Family history of breast cancer in first-degree relatives / BMI / Oral contraceptive use

Table 3 shows odds ratios by type of progestagen in combined EP therapy among current MHT users. The odds ratio for EP therapy containing natural micronized progesterone was below unity. It increased to 1.72 [1.11-2.65] for EP therapy containing synthetic progestagens, and a dose-response trend with duration of use was observed (p <0.01). Odds ratios for EP combinations containing testosterone-derivatives (3.35 [1.07-10.4]) were somewhat higher than for EP combinations with progesterone-derivatives (1.57 [0.99-2.49]). When using progesterone-derivatives users as baseline, the odds ratio for testosterone-derivative users was 1.81 [0.50-6.49] (not shown). The odds ratios associated with continuous and sequential regimens were 2.52 [0.77-8.32] and 1.75 [1.09-2.79], respectively. Out of 9 cases and 5 controls with continuous regimen, 8 cases and 4 controls used an EP combination containing a testosterone-derivative, making impossible the study of the specific effect of the progestagen and the regimen. 

**Table 3:** Odds ratios for breast cancer among current users of combined MHT by type of treatment and duration of use.

	**Any duration**				**Duration < 4 years**				**Duration ≥ 4 years**			
	**Cases**	**Controls**	**OR^a^**	**95% CI**	**Cases**	**Controls**	**OR^a^**	**95% CI**	**Cases**	**Controls**	**OR^a^**	**95% CI**
**Never MHT use**	311	357	1	ref	311	357	1	ref	311	357	1	ref
**Estrogen + natural progesterone**	25	34	0.80	[0.44-1.43]	10	17	0.69	[0.29-1.68]	14	17	0.79	[0.37-1.71]
**Estrogen + synthetic progestagen**	67	48	**1.72**	**[1.11-2.65]**	11	14	1.17	[0.48-2.86]	55	34	**2.07**	**[1.26-3.39]**
By type of synthetic progestagen												
*Estrogen + Progesterone Der.*	55	43	**1.57**	**[0.99-2.49]**	10	13	1.02	[0.40-2.58]	45	30	**1.92**	**[1.13-3.27]**
*Estrogen + Testosterone Der.*	11	5	**3.35**	**[1.07-10.4]**	4	4	1.64	[0.38-7.15]	7	1	**9.47**	**[1.09-82.6]**
By regimen												
*Continuous*	9	5	2.52	[0.77-8.32]	3	2	2.41	[0.36-16.1]	6	3	2.70	[0.60-12.2]
*Sequential*	56	40	**1.75**	**[1.09-2.79]**	11	10	1.40	[0.54-3.65]	45	30	**2.00**	**[1.18-3.41]**

a Odds Ratios adjusted for Study area/ Age at reference date / Age at menarche / Parity / Age at first full-term pregnancy / Breast feeding /History of benign Breast disease / Family history of breast cancer in first-degree relatives / BMI / Oral contraceptive use

Elevated odds ratios for EP therapy with synthetic progestagen and for tibolone were observed for ER-positive and for PR-positive tumors. Odds ratios were slightly smaller for ER and PR-negative cancers or did not reach statistical significance, but they are based on small numbers. Elevated odds ratios were observed for the two main histological types of breast cancer, with odds ratio of 5.87 for lobular carcinoma among tibolone users (table 4).

**Table 4:** Odds ratios for breast cancer among current users of menopausal hormone therapy by hormonal receptor status and histology.

		**Estrogen Receptor Status**					
	**Controls**	**ER positive cases**			**ER negative cases**		
	(n=816)	(n=590)			(n=99)		
	**N**	**N**	**OR^a^**	**95% CI**	**N**	**OR^a^**	**95% CI**
**Never MHT use**	357	242	1	ref	51	1	ref
**Estrogen-only therapy**	31	29	1.34	[0.76-2.37]	2	0.35	[0.07-1.54]
**Combined EP therapy**	82	70	1.36	[0.92-2.02]	15	0.97	[0.49-1.91]
*Estrogen+Natural progesterone*	*34*	*19*	*0.81*	*[0.43-1.54]*	*2*	*0.25*	*[0.05-1.17]*
*Estrogen+Synthetic progestagen*	*48*	*51*	***1.79***	***[1.12-2.86]***	*13*	*1.48*	*[0.71-3.10]*
**Tibolone**	8	13	2.57	[0.97-6.83]	1	0.83	[0.09-7.86]
		**Progesterone Receptor Status**					
	**Controls**	**PR positive cases**			**PR negative cases**		
	(n=816)	(n=462)			(n=219)		
	**N**	**N**	**OR^a^**	**95% CI**	**N**	**OR^a^**	**95% CI**
**Never MHT use**	357	189	1	ref	102	1	ref
**Estrogen-only therapy**	31	25	1.52	[0.84-2.76]	7	0.65	[0.27-1.57]
**Combined EP therapy**	82	54	1.35	[0.89-2.06]	30	1.17	[0.70-1.97]
*Estrogen+Natural progesterone*	*34*	*16*	*0.86*	*[0.44-1.70]*	*4*	*0.33*	*[0.11-1.01]*
*Estrogen+Synthetic progestagen*	*48*	*38*	***1.74***	***[1.05-2.88]***	*26*	*1.74*	*[0.98-3.10]*
**Tibolone**	8	10	**2.80**	**[0.99-7.89]**	4	1.51	[0.40-5.73]
		**Histology**					
	**Controls**	**Ductal**			**Lobular**		
	(n=816)	(n=586)			(n=125)		
	**N**	**N**	**OR^a^**	**95% CI**	**N**	**OR^a^**	**95% CI**
**Never MHT use**	357	239	1	ref	54	1	ref
**Estrogen-only therapy**	31	26	1.13	[0.63-2.01]	7	1.78	[0.70-4.54]
**Combined EP therapy**	82	75	1.34	[0.91-1.98]	13	1.56	[0.76-3.22]
*Estrogen+Natural progesterone*	*34*	*23*	*0.91*	*[0.50-1.67]*	*2*	*0.41*	*[0.09-1.87]*
*Estrogen+Synthetic progestagen*	*48*	*52*	***1.68***	***[1.05-2.67]***	*11*	***2.48***	***[1.12-5.52]***
**Tibolone**	8	11	1.93	[0.71-5.22]	6	**5.87**	**[1.66-20.7]**

a Odds Ratios adjusted for Study area/ Age at reference date/Age at menarche / Parity / Age at first full-term pregnancy / Breast feeding /History of benign Breast disease / Family history of breast cancer in first-degree relatives / BMI / Oral contraceptive use

Table 5 shows odds ratios by time interval between onset of menopause and first use of hormone therapy using never MHT users as baseline. 73% of current MHT users started treatment within the first year of onset of menopause, and had higher odds ratios than late MHT users who delayed treatment beyond one year. The odds ratio of early versus late users of EP therapy used as baseline was 2.96 [0.86-10.1] (not shown), and was not changed after adjustment for duration of use. 

**Table 5:** Odds ratios for breast cancer among current users of menopausal hormone therapy by time interval between start of menopause and start of MHT use^**a**^.

	**MHT started before or within one year after start of menopause**				**MHT started more than one year after start of menopause**			
	**Cases**	**Controls**	**OR^b^**	**95% CI**	**Cases**	**Controls**	**OR^b^**	**95% CI**
**Never MHT use**	311	357	1	ref	311	357	1	ref
**Estrogen-only therapy**	8	14	0.59	[0.22-1.58]	5	2	3.40	[0.60-19.3]
**Combined EP therapy**	52	38	**1.65**	**[1.02-2.69]**	14	16	1.05	[0.47-2.34]
*Estrogen+Natural progesterone*	10	15	0.74	[0.31-1.78]	5	8	0.72	[0.22-2.39]
*Estrogen+Synthetic progestagen*	42	23	**2.32**	**[1.30-4.12]**	9	8	1.43	[0.50-4.09]
**Tibolone**	4	2	2.09	[0.32-13.5]	6	-	-	-

a Analyses restricted to current users of MHT who used only one type of MHT

b Odds Ratios adjusted for study area / Age at reference date/ Age at menarche / Parity / Age at first full-term pregnancy / Breast feeding /History of benign breast disease / Family history of breast cancer in first degree relatives / BMI / Oral contraceptive use.

## Discussion

Our findings suggest that the type of progestagen in combined EP therapies used in France may modify the risk of breast cancer, and that tibolone, a molecule used as an alternative to EP therapy, increases risk. In addition, we found that women who start MHT early after onset of menopause were at increased risk as compared to women who delay treatment beyond one or more year. We also found that EP combinations were more strongly associated with ER positive than with ER negative tumors. 

We found no significantly increased risk of breast cancer in estrogen-only therapy users, a result in line with large observational cohort studies that reported small increased risks with hazard ratios in the range of 1.3-1.4 [13,22]. Contrasting with these findings, the WHI clinical trial reported that hysterectomized women treated with estrogens alone were at decreased risk of breast cancer [26]. These discrepancies between observational studies and clinical trials may be accounted for by different characteristics of the study populations [26,27]. 

While estradiol is virtually the sole estrogenic component of combined EP therapy in France [21], a large variety of progestogenic components has been used. Natural micronized progesterone is commonly used in France in EP combinations [18,21]. In our study, 25% of EP therapy users were treated with a combination of EP containing micronized progesterone, close to the proportion of 24% among French women of the E3N cohort study [13]. We found no indication of an increased risk of breast cancer in EP therapy users of micronized progesterone, a result in line with the finding of the E3N cohort [19,21]. Because micronized progesterone was rarely prescribed in countries other than France [13,18], the finding of the present study is the sole confirmation to date. This result however needs careful interpretation. First, the number of current users may have been too small in these studies to detect small increases in risk. Moreover, natural micronized progesterone might not oppose efficiently the estrogenic constituent of EP combinations, at least at the doses commonly used, and may thus provide poor protection against endometrial cancer. An increased risk of endometrial cancer in users of combined therapy containing natural progesterone was actually reported in the E3N-EPIC study [28]. Prescription of EP therapy containing natural micronized progesterone should thus be made with informed judgment in menopausal women.

Combined EP therapies that do not contain micronized progesterone were used by 42% of current MHT users in our study. Among these, the progestogenic constituent was a progesterone-derivative (90%) or a testosterone-derivative (10%). By contrast, testosterone-derivatives are more commonly used in Northern European countries [13,18,20]. In the present study, the odds ratio for breast cancer was greater for testosterone- (OR 2.7) than for progesterone- (OR 1.6) derivatives, although the difference did not reach statistical significance. It has been suggested that testosterone derivatives may be associated with a greater risk than EP combinations with progesterone derived progestagen due to indirect effects of testosterone derivatives stimulating breast cancer cells in synergy with estrogens or increasing estrogen bioavailability [29]. However, it has also been suggested that the higher risk might reflect a dose-response relationship rather than a real difference in progestogenic effect between progestagens [13,24]. In our study, the testosterone-derived progestagen was almost always administered continuously, a regimen that can provide 2 to 3 higher monthly dose of progestagen than a sequential regimen [13,30]. By contrast, the sequential regimen was usual in women treated with EP combinations containing a progesterone-derivative. It cannot be determined from our data if the difference in risk between progesterone and testosterone-derived progestagens is related to the type of progestagen itself or to the dose at which it is administered. 

Tibolone is a synthetic steroid hormone prescribed to menopausal women, as an alternative to classical MHT. Large epidemiologic studies reported that tibolone use was associated with an increased breast cancer risk [13,22,23,31], although smaller studies did not find an association [20,24,25]. In our study, we reported an elevated although non-significant increased risk of breast cancer among current users of tibolone based on a limited number of treated women. The increased risk of breast cancer associated with use of tibolone should be further scrutinized.

Stratification of breast cancer patients by receptor status (ER and PR) indicated that EP combinations was slightly stronger with ER and PR-positive than with ER and PR-negative tumors. EP combination containing synthetic progestagens was also more strongly associated with lobular than with ductal carcinoma. These results are consistent with the literature [32-34]. Interestingly, use of tibolone was also strongly associated with ER-positive and PR-positive tumors, and with lobular carcinomas. 

Several cohort studies have reported that the time interval between onset of the menopause and start of MHT treatment may influence breast cancer risk in menopausal women, with shorter treatment-free time interval being associated with higher risk [15-17]. In the present study, women who started using MHT within one year after onset of the menopause were at higher risk than women who delayed treatment beyond one year. The increased risk of breast cancer among early users of MHT hypothesized to be related to delayed lobular involution of the breast, a physiological age-related phenomenon increasing at menopause in untreated women that has been associated with a decreased risk of breast cancer [35]. Thus, delayed prescription of MHT in women starting menopause may help to decrease breast cancer risk. 

### Study strengths and limits

In this population-based study, we sought to include all incident cases diagnosed in the study populations during the study period. Cases were identified from active real-time search in the main cancer hospital of each area, and from smaller public and private hospitals, with a high participation rate. The number of eligible cases identified was close to the expected number based on age-specific incidence rates for France [36]. Controls were selected to reflect the distribution by socioeconomic status of the source populations. Differential participation rates between cases and controls across SES categories that could have distorted the association between MHT and cancer are therefore very unlikely. As in any case-control study, recall bias could not be excluded, but it was reduced in our study by the use of standardized questionnaires by trained interviewers, similar interview conditions for cases and controls, and the use of a validated questionnaire for eliciting information on MHT use. Our study was of relatively large size, and the statistical power was sufficient to detect an odds ratio of 1.38 for a prevalence of exposure among controls of 20% (the proportion of synthetic EP treatment users in the control group). However, the statistical power may have been limited in some stratified analyses. 

## Conclusion

One important finding is the absence of increased breast cancer risk in women using EP treatment containing natural progesterone, a formulation which is used by a large number of MHT treated women in France. However this type of treatment may not protect against endometrial cancer. We also suggested differences in breast cancer risk according to the type of synthetic progestagen in the EP treatment, with indications of higher risks associated with testosterone derivatives that may be related to the dose rather than to the type of progestagen itself. Our results also indicate that early users of MHT after start of menopause may further increase their risk of breast cancer. Pooled analyses of several studies conducted in France may help to further scrutinize the associations between type of MHT and breast cancer. 
